# Meta-Analysis Identifies NF-κB as a Therapeutic Target in Renal Cancer

**DOI:** 10.1371/journal.pone.0076746

**Published:** 2013-10-07

**Authors:** Suraj Peri, Karthik Devarajan, Dong-Hua Yang, Alfred G. Knudson, Siddharth Balachandran

**Affiliations:** 1 Department of Biostatistics and Bioinformatics, Fox Chase Cancer Center, Philadelphia, Pennsylvania, United States of America; 2 Biosample Repository Core Facility, Fox Chase Cancer Center, Philadelphia, Pennsylvania, United States of America; 3 Cancer Biology Program, Fox Chase Cancer Center, Philadelphia, Pennsylvania, United States of America; 4 Immune Cell Development and Host Defense Program, Fox Chase Cancer Center, Philadelphia, Pennsylvania, United States of America; Johns Hopkins School of Medicine, United States of America

## Abstract

**Objective:**

To determine the expression patterns of NF-κB regulators and target genes in clear cell renal cell carcinoma (ccRCC), their correlation with von Hippel Lindau (VHL) mutational status, and their association with survival outcomes.

**Methods:**

Meta-analyses were carried out on published ccRCC gene expression datasets by RankProd, a non-parametric statistical method. DEGs with a False Discovery Rate of < 0.05 by this method were considered significant, and intersected with a curated list of NF-κB regulators and targets to determine the nature and extent of NF-κB deregulation in ccRCC.

**Results:**

A highly-disproportionate fraction (~40%; *p* < 0.001) of NF-κB regulators and target genes were found to be up-regulated in ccRCC, indicative of elevated NF-κB activity in this cancer. A subset of these genes, comprising a key NF-κB regulator (*IKBKB*) and established mediators of the NF-κB cell-survival and pro-inflammatory responses (*MMP9*, *PSMB9*, and *SOD2*), correlated with higher relative risk, poorer prognosis, and reduced overall patient survival. Surprisingly, levels of several interferon regulatory factors (IRFs) and interferon target genes were also elevated in ccRCC, indicating that an ‘interferon signature’ may represent a novel feature of this disease. Loss of VHL gene expression correlated strongly with the appearance of NF-κB- and interferon gene signatures in both familial and sporadic cases of ccRCC. As NF-κB controls expression of key interferon signaling nodes, our results suggest a causal link between VHL loss, elevated NF-κB activity, and the appearance of an interferon signature during ccRCC tumorigenesis.

**Conclusions:**

These findings identify NF-κB and interferon signatures as clinical features of ccRCC, provide strong rationale for the incorporation of NF-κB inhibitors and/or and the exploitation of interferon signaling in the treatment of ccRCC, and supply new NF-κB targets for potential therapeutic intervention in this currently-incurable malignancy.

## Introduction

Renal cell carcinomas (RCC) account for about 3% of all adult cancers, and cause ~116,000 annual worldwide deaths [1]. Several histological sub-types of RCC have been described, including chromophobe, papillary and clear cell, of which the clear cell variant (ccRCC) accounts for ~85% of all RCC cases [2,3]. Early-stage ccRCC is usually curable by surgery, and patients diagnosed with localized renal masses of < 4 cm. have excellent prognosis [4]. ccRCC is, however, a largely asymptomatic disease, and approximately one-third of all patients present with locally-advanced or metastatic cancer at the time of diagnosis. By contrast with localized early-stage ccRCC, advanced ccRCC is a lethal, chemotherapy-resistant cancer [1,5].

Advanced ccRCC is treated primarily by small-molecule pharmacological approaches. Frontline options include the tyrosine kinase inhibitors sunitinib and sorafenib, and the mTOR inhibitors temsirolimus and everolimus. These agents, however, only provide short-term benefit by delaying disease progression, and are not curative [6–8]. Moreover, such agents require continuous administration, exposing patients to significant side-effects [7,9]. Treatment of metastatic RCC is therefore still a therapeutic challenge in need of new options.

A genetic hallmark of ccRCC is inactivation of the von Hippel Lindau (VHL) tumor suppressor gene [10]. The *VHL* gene is inactivated by either mutation or hypermethylation in up to 90% of sporadic ccRCC cases [10–12]. In its best-described role, pVHL, the product of the *VHL* gene, functions as part of a degradative E3 ubiquitin ligase complex that tightly controls protein levels of Hypoxia Inducible Factor (HIF), a transcription factor and master regulator of the cellular response to hypoxia [11,13]. When pVHL is absent, HIF accumulates even under normoxic conditions, and inappropriately transactivates expression of its target genes. As many HIF targets are potently tumorigenic, mis-expression of HIF target genes are considered the primary orchestrators of *VHL*-deficient ccRCC tumor progression, and targeting pathways downstream of HIF (e.g. VEGF signaling) represents a primary pharmacological approach to treating RCC [11].

In addition to regulating HIF, pVHL has been shown in several cell culture studies to also control activity of the transcription factor NF-κB [14]. When pVHL expression is lost (or ablated by RNAi), NF-κB activity is elevated; conversely, re-introduction of pVHL into *VHL*-null RCC cells lowers NF-κB activity [15–17]. These observations have important clinical ramifications. First, as NF-κB (like HIF) is a central regulator of inflammatory and cell-survival responses, it is very likely that elevated NF-κB signaling following pVHL loss will contribute to steps in the genesis, progression, survival, and/or spread of ccRCC. Second, if NF-κB is in fact elevated in ccRCC tumors – and not just in RCC cell lines - then targeting NF-κB provides an exciting new therapeutic option for advanced ccRCC. In this regard, initial studies have shown that small-molecule inhibition of NF-κB sensitizes otherwise-resistant ccRCC cells to (1) the tumoricidal activity of EGFR inhibitors, (2) apoptosis by the anti-tumor cytokine TRAIL, and (3) oncolysis by encephalomyocarditis virus [14,18–21]. For these reasons, obtaining insight into the nature and extent of NF-κB de-regulation in ccRCC tumors becomes an important objective.

We initiated this study to determine through large-scale bioinformatic approaches the prevalence of NF-κB transcriptomic deregulation in patient-derived ccRCC samples. From these analyses, we have found that NF-κB appears to be constitutively active in a high percentage of ccRCC cases, and that a disproportionate number of NF-κB regulators and targets (the ccRCC ‘NF-κB signature’) display consistently elevated expression in ccRCC, compared to normal renal tissue. Further investigation also revealed the presence of a robust ‘interferon (IFN) signature’ in ccRCC. We show that the appearance of both NF-κB and IFN signatures are well-correlated with VHL mutational status, and identify a key subset of NF-κB regulators and targets whose elevated expression correlates with higher relative-risk, poorer prognosis, and reduced overall survival in ccRCC. Collectively, these results indicate that elevated NF-κB and IFN signaling may represent common features of pVHL-negative ccRCC, and provide rationale for targeting NF-κB in this disease.

## Materials and Methods

### Ethics statement

The use of human tissue samples from patients at the Fox Chase Cancer Center was approved by the Fox, Chase Institutional Review Board. Written informed consent, approved by the ethics committee, was obtained for the use of these samples.

### Immunohistochemistry

A kidney tissue microarray (TMA), containing duplicate slices from 20 ccRCC tumors and 8 normal kidneys, was constructed from archival formalin fixed, paraffin-embedded Fox Chase Cancer Center patient samples. TMA tissue sections were cut with a thickness of 5 microns, deparaffinized by xylene and rehydrated in decreasing concentration of ethanol. Antigen retrieval was achieved by boiling sections on 10mM citrate buffer for 20 minutes. After blocking of endogenous peroxidase with 3% hydrogen peroxidase in methanol, sections were incubated with Background Sniper (Biocare Medical) at room temperature for 30 minutes. The sections were next incubated with primary antibodies, NF-κB (Cell Signaling) at the dilution of 1:500, and STAT1 (BD Biosciences) at a dilution of 1:100, at 4°C overnight. After washing in PBS, sections were incubated with Labeled Polymer-HRP anti-rabbit and anti-mouse (DAKO) secondary antibody at RT for 1h, exposed to diaminobenzidine tetrahydrochloride solution, and counterstained with hematoxylin. After dehydrating in increasing concentrations of ethanol and clearing in xylene, sections were mounted in Permount. Images were taken on a Nikon Eclipse E600 microscope with NIS Elements D3.0 software.

### Meta-analysis

For meta-analyses, we compiled a list of published RCC studies from Gene expression Omnibus (GEO) or ArrayExpress (Table S1), whose data were (i) generated using Affymetrix platforms U133A, U133B, and U133Plus2; (ii) accurately annotated when deposited into databases; and (iii) contained normal tissue controls. For studies employing the U133A and U133B chips, we only considered those which profiled samples on *both* chips; this maximized the number of genes available for subsequent meta-analysis. Raw data were normalized using Robust Multi-array Average (RMA) [22]. In cases where samples were profiled on two different platforms (e.g. Affymetrix U133A and U133B), probe sets with higher mean expression values were selected if multiple probe sets mapped to same gene. The datasets were then merged based on gene symbol using the MergeMaid package (http://astor.som.jhmi.edu/MergeMaid) available through Bioconductor [23]. The meta-analyses were carried out using the RankProd method [24], a non-parametric statistical method, that utlilzes ranks of differentially expressed genes (DEGs) among the different studies to generate a list of DEGs between two conditions (for example, ccRCC vs. normal). The significance of differential gene-expression is then calculated based on percentage of false positive predictions (i.e. the False Discovery Rate, or FDR). For this study, we selected our lists of DEGs based on an FDR of 0.05 (5%) calculated based on 10,000 permutations. To define the NF-κB and IFN signatures, curated NF-κB and IFN genes were intersected with up-regulated DEGs. To examine NF-κB and IFN signatures in samples with mono- or biallelic inactivation of *VHL*, DEGs were calculated using LIMMA [25] and RMA-normalized data. Our methodology is summarized in the flowchart presented in Figure S1.

### Survival analysis

Gene expression and survival data available for 55 ccRCC patients in the TCGA database (https://tcga-data.nci.nih.gov/tcga/) was used for survival analyses using univariate Cox proportional hazards (PH) and Accelerated Failure Time (AFT) models [26]. A goodness-of-fit (GOF) test of the Cox PH model was performed [27]. While the Cox PH model implicitly assumes that the hazard and survival curves corresponding to two different values of a covariate do not cross, the AFT model allows crossing of curves [28,29] and accounts for non-proportionality of hazards (or risk of death) in the two groups. All tests were two-sided and used a Type I Error of 0.05 to determine statistical significance. In addition to the *p*-values for each model-fit, estimates of relative risk (RR) from the Cox PH and coefficient (β) from the AFT models, respectively, were used to determine the magnitude of association between gene expression and overall survival. An RR estimate in excess of 1 or a negative estimate of β indicate poor prognosis with increasing expression. To visualize the association of gene expression levels with overall survival, individual gene expression profiles were dichotomized by median split into ‘high’ or ‘low’ expression groups, and Kaplan-Meier survival curves were plotted for each group. Computations were done using the packages *survival* and *lss* in the R statistical language and environment (http://www.r-project.org). 

## Results

### Meta-analysis identifies NF-κB deregulation in ccRCC

**Figure 1:**
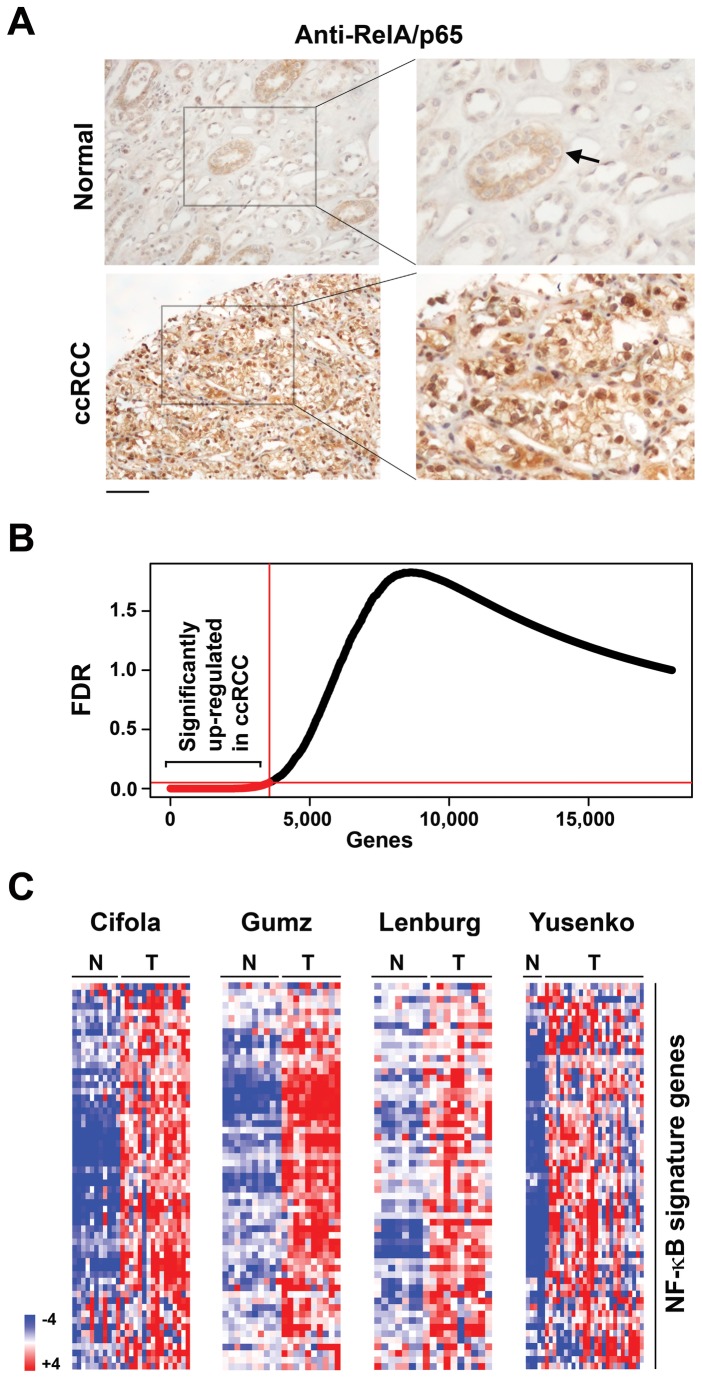
An NF-κB signature in **ccRCC** . (a) Immuno-histochemical staining showing prominent nuclear RelA signal in ccRCC samples, but not in normal kidney tissue. The arrow indicates cytoplasmic RelA staining in cells of the proximal tubular epithelium. Scale bar = 100 µM. (b) Up-regulated genes (X-axis) from the meta-analysis of the four ccRCC datasets were plotted against false-discovery rate (FDR, Y-axis). Up-regulated genes with FDR < 0.05, shown in red, were used to define NF-κB and IFN signatures. (c) Heatmaps showing expression of NF-κB signature genes in each of the four indicated studies. N = normal, T = tumor. Heat bar = expression levels (log_2_ scale).

While examining a publicly-available DNA microarray dataset of ccRCC and paired normal samples [30,31], we noticed that several established NF-κB target gene mRNAs were over-expressed in ccRCC samples, compared to their normal controls. Given the therapeutic ramifications of these observations, we sought to determine if NF-κB is constitutively active in ccRCC. We therefore examined patient-derived ccRCC specimens for nuclear localization of the classical NF-κB sub-unit RelA/p65. We focused on RelA/p65, as previous studies have shown that RelA-containing dimeric complexes are the dominant form of NF-κB in ccRCC cell lines, and that these complexes re-localize from the cytoplasm to the nucleus when active [32–34]. Out of 20 distinct ccRCC specimens examined, 16 (80%) displayed robust nuclear staining of RelA in >50% of cells, indicative of constitutive NF-κB activity in these cells. A further two cases showed weak nuclear staining in <20% of cells, and two others manifested no detectable RelA signal in the nucleus. By contrast, none (0/8) of the normal renal sections examined displayed detectable nuclear RelA staining. A typical example of intense nuclear RelA staining in ccRCC – but not normal kidney tissue - is shown in Figure 1a; note that normal cells of the proximal tubular epithelium, from which ccRCC is thought to arise, show evidence of *cytoplasmic* RelA (Figure 1a, arrows). These results suggest that constitutively-active nuclear NF-κB may be a common feature in ccRCC, perhaps as a consequence of NF-κB activation in the tubular epithelium during RCC tumorigenesis.

To investigate the extent of NF-κB target-gene deregulation in ccRCC, we first defined a list of genes whose expression is known to regulate and/or be regulated by NF-κB. Reasoning that aberrant NF-κB activity will be reflected in the altered expression of these genes, we combined a publicly-available list of annotated NF-κB target genes [(HUhttp://bioinfolifl.fr/NF-KBUH, based largely on [35]] with our own datasets [36,37] to curate a total of 137 genes (Table S2) that are known to regulate NF-κB, whose promoters contain putative/validated NF-κB binding sites, and/or whose expression has been shown to rely on NF-κB activity in various contexts.

We next examined by meta-analysis the expression profiles of these 137 NF-κB target genes in whole-genome transcriptomic data from 61 ccRCC and 34 normal samples across four independent studies that we refer to here by the names of their first authors: Cifola, Gumz, Lenburg and Yusenko [31,38–40]. Criteria for selection of these studies are summarized in Figure S1. For this meta-analysis, we used RankProd, a non-parametric statistical method capable not only of integrating data from a variety of platforms, but also of handling experimental variability between datasets [24]. Of ~18,000 total genes examined, 3,560 were found to be uniformly up-regulated in ccRCC (Figure 1b), while 2,797 genes were consistently down-regulated, at a false-discovery rate (FDR) of ≤ 0.05. Of these, 58 genes (~42% of all curated NF-κB targets) were up-regulated in ccRCC samples, compared to normal controls. The ratio of NF-κB genes up-regulated in ccRCC (58/137) is highly significant, (*p*-value < 0.001, one-tailed proportion *Z*-test), when compared to the percentage of all genes up-regulated in ccRCC (3560/17997; ~20%). By contrast, three-fold fewer NF-κB target genes (18 genes, representing 13% of NF-κB targets) were down-regulated in ccRCC; this was found not to be significant (p-value = 0.74). The results from this analysis indicate that ccRCC specimens display selective, uniformly-elevated expression of a subset of NF-κB target genes. We designate these genes the ccRCC ‘NF-κB gene signature’. Figure 1c depicts the expression profiles of the NF-κB gene signature in each of the four studies.

**Figure 2:**
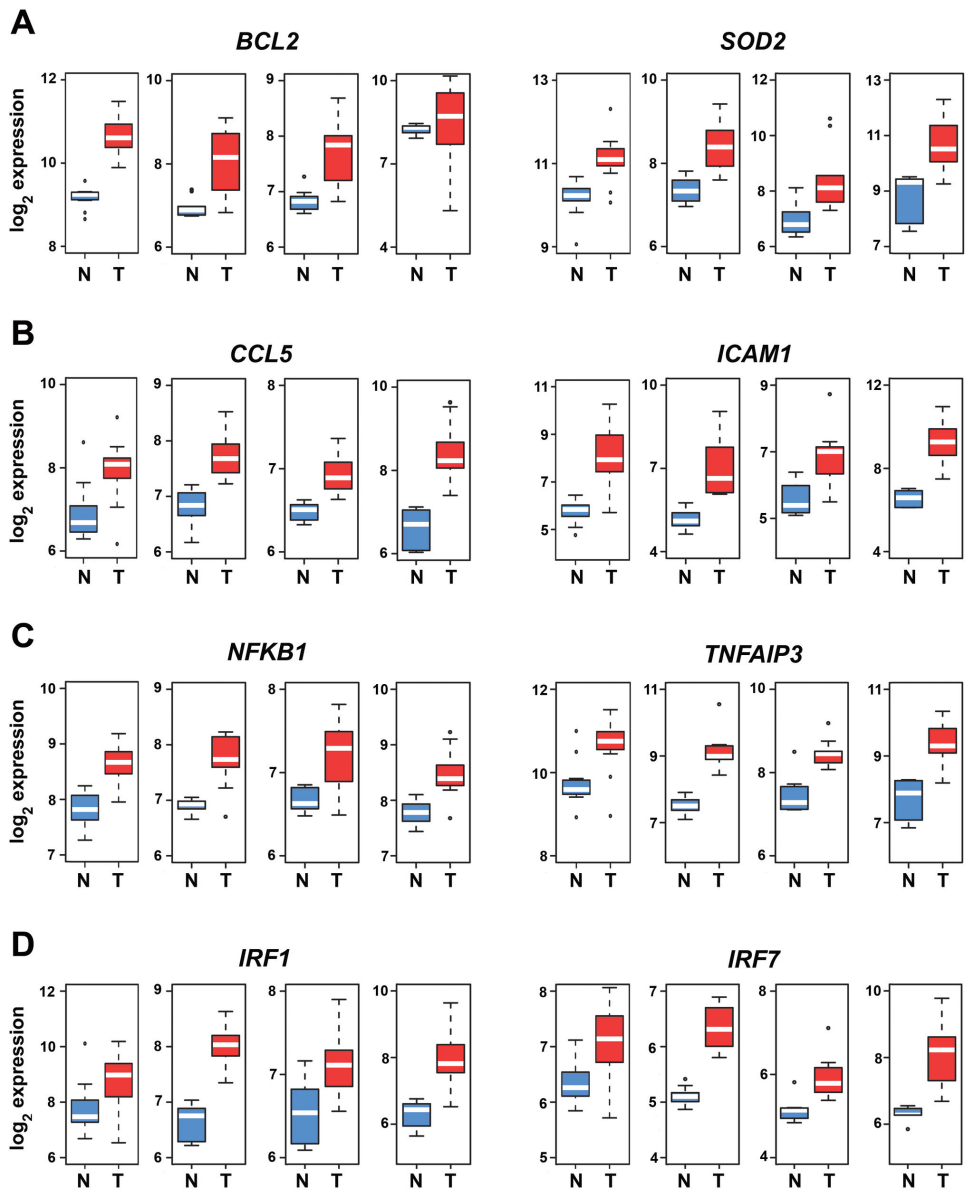
Box plots depicting individual mRNA expression levels of representative genes from the NF-κB signature. (a) Cell-survival genes *BCL2* and *SOD2*, (b) pro-inflammatory genes *CCL5* and *ICAM1*, (c) NF-κB regulators *NFKB1* and *TNFAIP3* and (d) interferon regulatory factors *IRF1* and *IRF7*. Data were normalized using RMA. Fold-changes for Tumor (T) versus Normal (N) comparison were obtained by LIMMA. Y-axis shows RMA-normalized expression level (on log_2_ scale) of each mRNA. Blue boxes represent gene expression levels in normal tissue, and red depicts expression in ccRCC. The white line within each box is the median, and distance between box and whiskers indicate interquartile ranges. Each of the four graphs per gene represent expression profiles in arrays generated by (from left to right) the Cifola, Gumz, Lenburg, and Yusenko studies.

The ccRCC NF-κB gene signature was sortable into four distinct categories: pro-inflammatory, cell-survival, NF-κB regulators, and, surprisingly, interferon regulators (Table S3). The majority of up-regulated NF-κB targets were pro-inflammatory (43/58). Of the remaining genes, seven were involved in cell survival, and five were feed-forward or feed-back regulators of the NF-κB response itself. Unexpectedly, three NF-κB targets (*IRF1, IRF2*, and *IRF7*) consistently up-regulated across all ccRCC specimens encoded interferon regulatory factors (IRFs), a family of transcription factors typically associated with the interferon-mediated innate-immune response to microbial infections [41]. Individual expression profiles of two representative genes from each category are shown in Figure 2. These results identify within the ccRCC NF-κB signature several well-established mediators of the NF-κB pro-inflammatory and cell-survival responses, as well as an unanticipated subset of IFN regulators.

### An interferon signature in ccRCC

Intrigued by the observation that IRF-encoding genes were up-regulated in ccRCC, we hypothesized that, in addition to elevated NF-κB activity, ccRCC cells likely display increased tonic type I (α/β) IFN signaling. Three published observations underlie this hypothesis. First, the genes encoding IRFs 1,2, and 7, in addition to being NF-κB targets, are also well-described IFN-stimulated genes (ISGs) [42,43]. Second, most cells maintain low levels of autocrine type I (α/β) IFN signaling, ostensibly in preparation for acute virus infections [44–46]. Third, we have previously reported that constitutive NF-κB signaling is necessary for maintenance of autocrine IFN signaling [36,44]. Together, these observations allow us to propose a model in which elevated NF-κB signaling ‘ramps up’ tonic type I IFN signaling, which then increases expression of ISGs (such as *IRFs*) in ccRCC.

**Figure 3:**
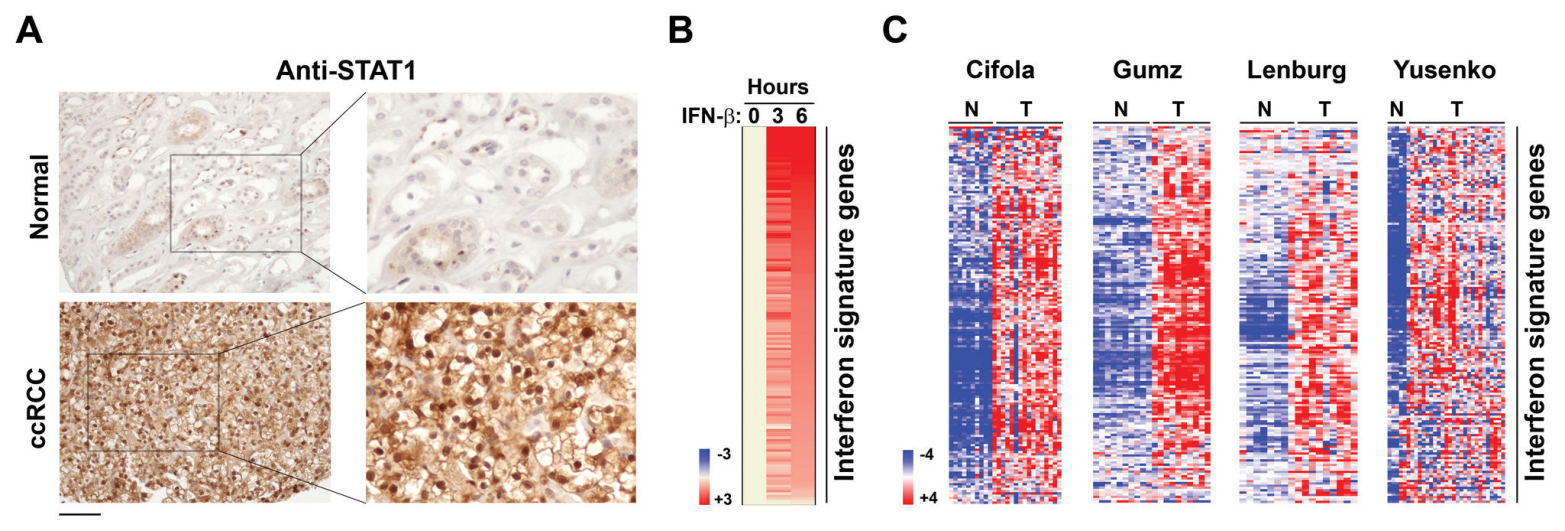
An IFN signature in **ccRCC** . (a) Immuno-histochemical staining showing robust nuclear STAT1 signal in ccRCC samples, but not in normal kidney tissue. (b) Heatmap showing expression of IFN signature genes after IFN stimulation of murine embryo fibroblasts. Heat bar = fold-change (log_2_ scale). Expression levels of untreated cells were arbitrarily set to 1 (beige). (c) Heatmaps showing expression of IFN signature genes in each of the four indicated studies. N = normal, T = tumor. Heat bar = expression levels (log_2_ scale).

To test this model, we examined RCC samples for hyperactive tonic type I IFN signaling. As direct measurement of tonic type I IFN levels in uninfected tissue is challenging and unreliable [36], we instead examined a downstream consequence of active IFN: nuclear localization of the key IFN-responsive transcription factor STAT1. Like NF-κB itself, STAT1 is normally cytoplasmic when inactive, but quickly translocates to the nucleus to drive ISG expression upon IFN stimulation [47]. If IFN signaling is constitutively elevated in RCC, the STAT1 will be expected to localize to the nucleus in RCC – but not normal – tissue. We found that 16/20 RCC samples, but none of the normal kidney specimens (0/8) – displayed strong nuclear STAT1 staining (Figure 3a). Remarkably, and in agreement with a causal link between elevated NF-κB signaling and increased tonic IFN activity, fully 100% of nuclear RelA-positive ccRCC samples were also positive for nuclear STAT1.

We identified from our previous work [36] a total of ~ 400 genes that are induced at least two-fold by type I IFNs (Figure 3b), for which expression data were also present in the four ccRCC studies. When we examined the expression of these ISGs in ccRCC datasets, a total of 164 ISGs were found to be up-regulated in ccRCC (~40% of all tested ISGs, p-value < 0.001 by one-tailed proportion Z-test), while only 51 ISGs were down regulated [~13%, p-value = 0.94]. These results indicate that, like with NF-κB, constitutively-elevated type I IFN signaling occurs in ccRCC. The 164-gene ‘IFN gene signature’ is listed in Table S4, and its expression profile in each of the four individual studies is shown in Figure 3c.

**Figure 4:**
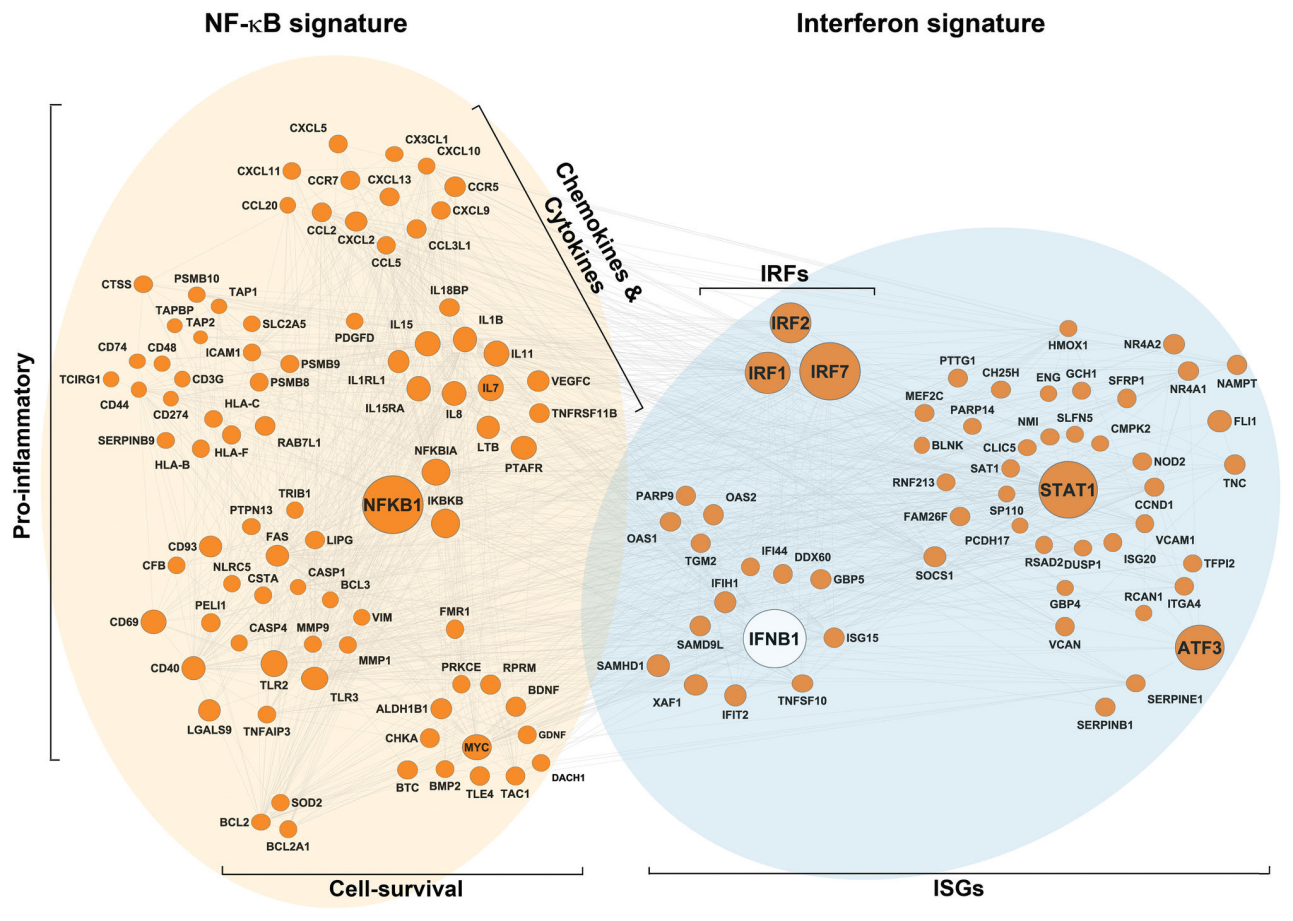
Molecular network of NF-κB and IFN signatures in **ccRCC** . The network was constructed by Ingenuity Pathway Analysis software (Ingenuity® Systems, HUhttp://www.ingenuity.comUH) using all genes in the NF-κB and IFN signatures. Key clusters in NF-κB (brown) and IFN (blue) arms are identified, and regulatory molecules controlling gene-network nodes are shown as large circles.

We next used Ingenuity Pathways analysis to construct a network that would identify inter-molecular relationships between NF-κB and IFN gene signatures. This analysis revealed robust connectivity between NF-κB and IFN signatures via IFN-β (encoded by *IFNB1*) and IRF nodes (Figure 4). Expectedly, pro-inflammatory and cell-survival clusters were observed within the NF-κB arm of the network, while numerous well-established innate-immune mediators were represented in the IFN signature arm (Figure 4). Taken together, these results support a causal link between NF-κB and IFN signatures mediated by autocrine IFN/IRF signaling.

### VHL mutational status correlates with expression of NF-κB and IFN signatures

**Figure 5:**
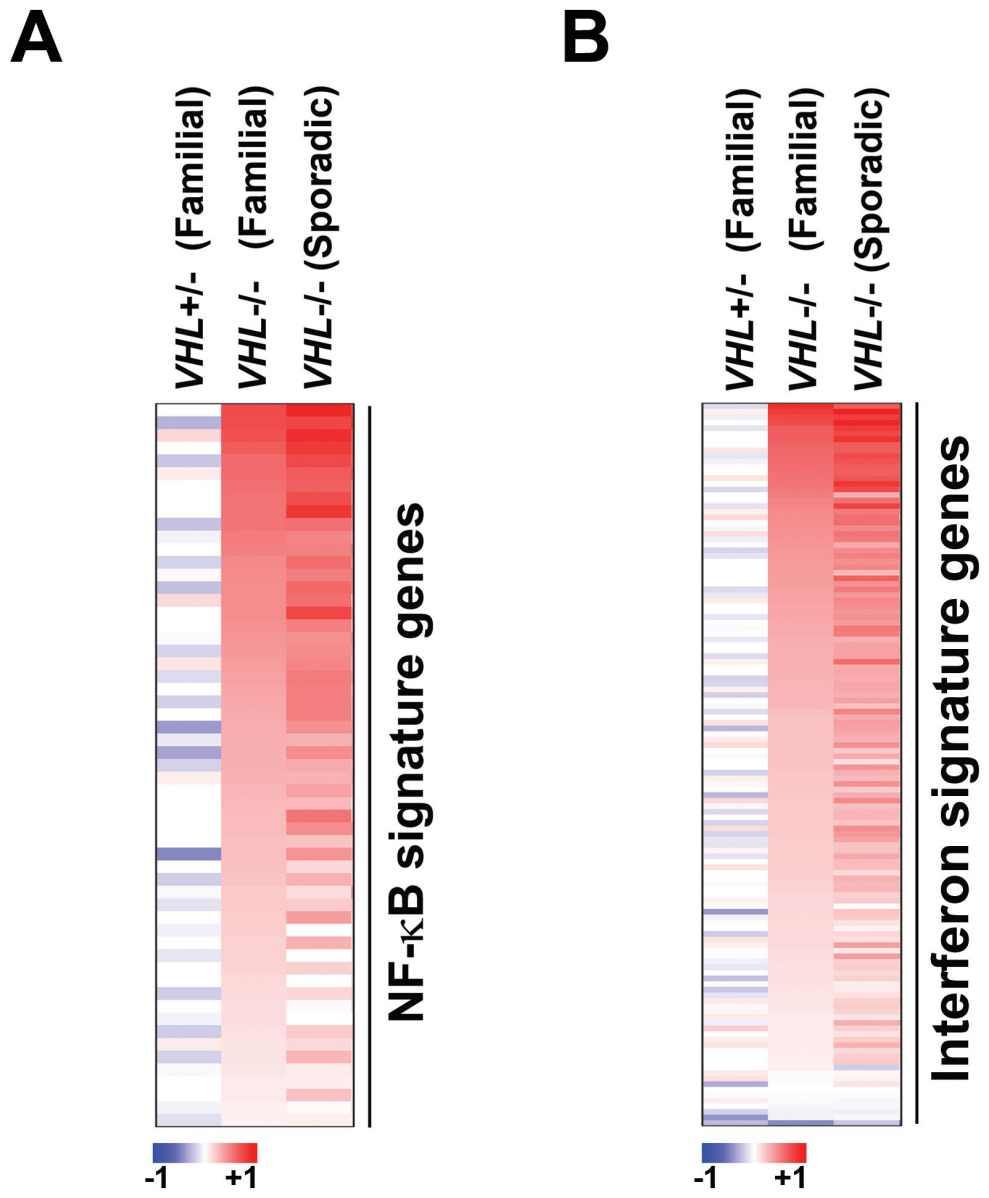
VHL mutational status correlates with expression of NF-κB and IFN signatures. Heatmaps showing fold-changes in expression levels of NF-κB signature genes (a) or IFN signature genes (b) between VHL+/- samples from cases of familial VHL disease compared to *VHL* +/+ normal renal epithelium (column 1) ; VHL-/- cases of familial ccRCC compared to normal renal tissue (column 2) ; or VHL-/- cases of sporadic ccRCC compared to normal renal tissue (column 3). Heat bar = fold-change (log_2_ scale).

Results from cell-culture studies suggest a causal link between the absence of functional pVHL protein and elevated NF-κB activity in ccRCC [15–17]. Given these observations, we inquired if the mutational status of *VHL* correlated with the appearance of NF-κB and IFN signatures in ccRCC. For this analysis, we compared to their respective normal controls (1) epithelial cell cultures of pre-neoplastic renal lesions from six familial cases of VHL patients harboring one functional copy of *VHL* [48], (2) ccRCC tissue from 32 familial cases of biallelically-inactivated *VHL* [49], and (3) ccRCC tissue from 20 sporadic cases of biallelically-inactivated *VHL* [49]. We found that neither NF-κB nor IFN signatures were present in patients with one functional copy of *VHL*. By contrast, ccRCC samples harboring biallelic loss of VHL (whether familial or sporadic in origin) displayed robust expression of both NF-κB and IFN signatures (Figure 5). These data provide strong support to the idea that VHL inactivation is likely causally linked to the appearance of elevated NF-κB and IFN activity in ccRCC.

### Elevated expression of subset of NF-κB targets correlate with poor outcome in ccRCC patients

**Figure 6:**
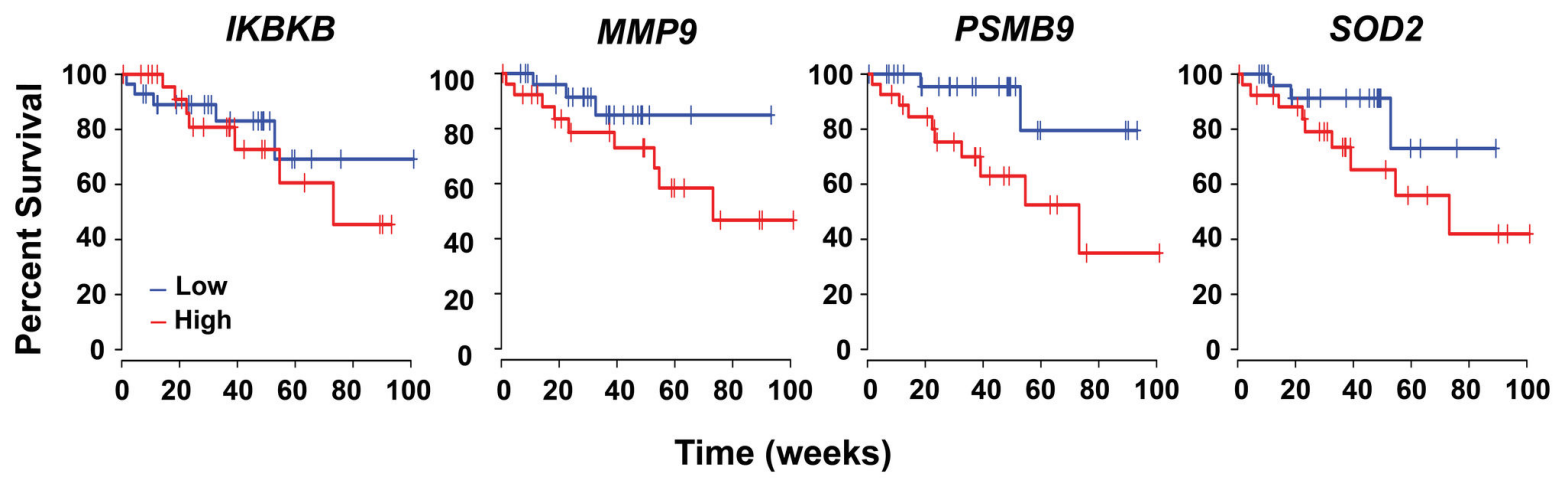
A subset of NF-κB regulators and target genes correlate with poor outcome in **ccRCC** . Kaplan-Meier survival curves for genes in the NF-κB signature whose increased expression levels significantly correlate with poorer overall survival outcome are shown. Individual gene expression profiles were dichotomized by median split into ‘high’ (red) or ‘low’ (blue) expression groups. Gene names are indicated above each graph. Please see Table S5 for *p*-values.

To determine if increased NF-κB activity was associated with poor survival outcomes in ccRCC, we examined the correlation between expression of genes in our NF-κB signature and overall survival for 55 ccRCC patients whose gene expression and survival data were available in The Cancer Genome Atlas (TCGA). From this analysis, we found that elevated expression of four NF-κB regulators and target genes (*IKBKB*, *MMP9, PSMB9*, and *SOD2*) was significantly associated with higher relative-risk (RR), poorer prognosis, and reduced overall patient survival by the Cox PH model (*p*-value <0.05, RR > 1) or by the AFT model (*p*-value <0.05 or β coefficient < 0; see Methods). These four genes comprise a key regulator of NF-κB signaling itself (*IKBKB*) and established mediators of the NF-κB cell-survival and pro-inflammatory responses (*MMP9, PSMB9*, and *SOD2*), raising the exciting possibility that selectively targeting members of this subset will have clinical benefit in ccRCC. Figure 6 presents the Kaplan-Meier curves for these genes and Table S5 summarizes the results of these analyses.

Of note, increased expression of a fifth gene (*NFKB1*, encoding the NF-κB sub-unit p105/p50) was also significantly associated with poorer overall survival by the AFT model (*p*-value=0.041). However, a positive-value β coefficient (33.6) and lack of significance by the Cox PH model (p-value = 0.41, RR = 0.5) precluded us from clarifying its association with ccRCC progression, for which reason we focused on *IKBKB*, *MMP9, PSMB9*, and *SOD2*.

## Discussion

In this study, we have determined that NF-κB is constitutively active in the majority of ccRCC samples tested, and have shown by meta-analysis that key NF-κB regulators and targets are uniformly up-regulated across four independent studies. We also report the discovery of an IFN signature in ccRCC, and provide compelling evidence that loss of VHL function is necessary for both NF-κB and IFN signatures. Finally, we identify a subset of potentially-druggable NF-κB regulators and targets whose elevated expression correlates with poor prognosis and survival. Of note, unavailability of patient data precluded us from examining if the NF-κB and/or IFN signatures correlated with ccRCC stage/grade.

Although NF-κB-mediated survival signaling likely evolved to protect cells from mitochondrial flux inherent to normal physiological responses (e.g. during cytokine-driven anti-microbial responses), several observations make it plausible that the NF-κB cell-survival response has been usurped by tumor cells to promote their own viability. For example, the founding member of the NF-κB family - the avian retroviral gene *v-Rel* – is a *bona fide* oncogene, and genes encoding NF-κB subunits and signaling components display activating mutations in several tumors [reviewed in [50–52]]. NF-κB cell-survival targets encode antioxidant enzymes that buffer mitochondria during times of increased bioenergetic demand, as well as other proteins (such as the Bcl-2 family members Bcl-X_L_ and Bfl-1) that actively prevent mitochondria from inducing cell death during genotoxic and metabolic stresses inherent to the process of tumorigenesis [51,52].

Loss of pVHL has been shown to result in increased NF-κB activity, indicating that activation of NF-κB may represent a common downstream consequence of *VHL*-deficiency [15–17,33]. The Kaelin and Rettig laboratories have provided mechanistic insight into how pVHL deficiency results in increased NF-κB activity by elucidating two distinct pathways of pVHL-dependent NF-κB regulation. Kaelin and colleagues identified the NF-κB activator CARD9 as a pVHL-interacting protein, and demonstrated that pVHL promoted inhibitory phosphorylation of CARD9 by the kinase CK2. Ablating pVHL expression increased CARD9-driven NF-κB activity, while abolishing CARD9 expression normalized NF-κB activity in pVHL-deficient RCC settings [17]. In parallel, An and Rettig determined that loss of pVHL, through a HIF → EGFR autocrine loop, results in increased NF-κB activity in ccRCC [16]. Both mechanisms connect pVHL deficiency to elevated NF-κB activity and provide biochemical explanations for our observation that heightened NF-κB correlates well with VHL mutational status.

**Figure 7:**
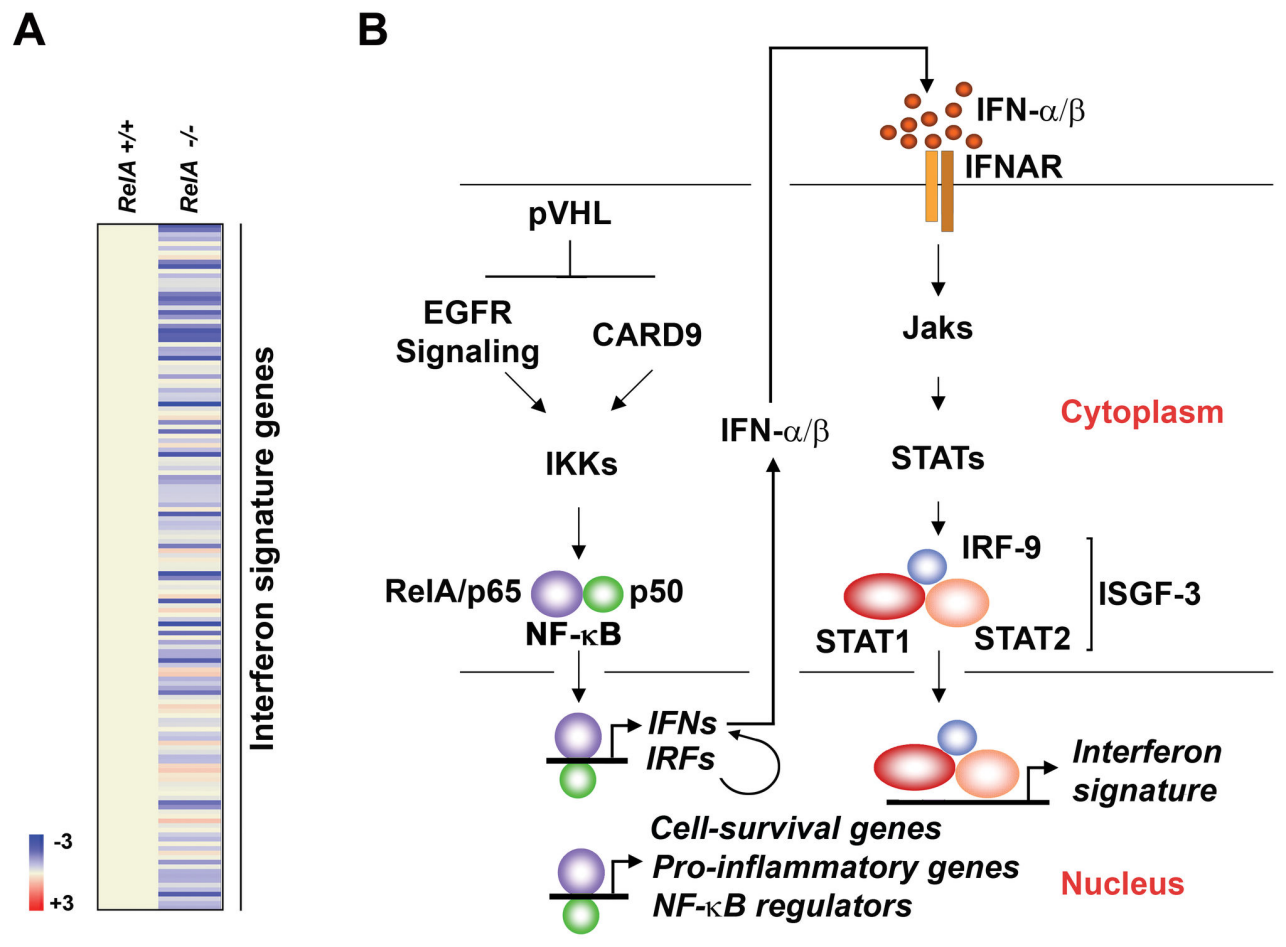
Model linking pVHL loss to NF-κB and IFN gene signatures. (a) Heatmap showing basal expression of the IFN gene signature in RelA+/+ and RelA-/- MEFs. Heat bar = fold-change (log_2_ scale). Expression levels in RelA+/+ were arbitrarily set to 1 (beige). Note that ~80% of IFN signature genes are expressed at lower basal levels in cells lacking RelA than in controls, indicative of a role for NF-κB in controlling ISG expression. (b) Schematic depicting the model presented in this study. ccRCC cells display elevated levels of NF-κB activity, perhaps as a result of result of constitutive EGFR or CARD9 activity stemming from loss of pVHL, that drives expression of cell-survival, pro-inflammatory, NF-κB regulatory genes, as well as genes encoding key nodes of type I interferon signaling (IFN-β, IRFs). Type I IFNs produced in this manner then function in an autocrine fashion to induce the expression of an IFN gene signature.

An unexpected result from this study was the discovery that an IFN signature characterizes ccRCC. The simplest explanation for this signature is that it is the direct consequence of elevated NF-κB activity. We favor this explanation for the reasons that (1) genes encoding key nodes of the IFN system, including IFN-β and IRF-7, contain functional NF-κB sites in their promoters [53,54], and simple activation of NF-κB can drive these promoters [43,54]; (2) a 1:1 correlation was found between ccRCC samples that contained nuclear NF-κB and those that displayed activated STAT1, and (3) the IFN signature collapses in cells lacking the NF-κB subunit RelA (Figure 7a). Based on these observations, we suggest that elevated NF-κB activity in ccRCC directly induces the IFN signature by interacting with NF-κB elements in the *IFNB1* and *IRF7* genes to stimulate their expression. IFN-β, and perhaps IRF-7-driven IFN-α subtypes [55] - produced in this manner would then act on surrounding cells to generate an IFN transcriptional signature (Figure 7b).

We considered two other explanations for an IFN signature in RCC, before settling on the one provided above. First, we evaluated the possibility that the IFN signature might simply be induced by residual recombinant IFN in the tumor samples as result of an IFN-based therapeutic regimen for these RCC patients. We discounted this possibility for two reasons: (1) an IFN-signature is seen in early-stage RCC samples [31], but IFN is not usually administered to RCC patients with localized disease, and (2) the specimens in our studies were obtained immediately post-surgery, but IFN is typically administered only after surgical removal of the primary tumor. Second, we speculated that infiltrating immune cells (such as NK cells, which can produce high levels of IFNs) might induce IFN gene expression in surrounding RCC cells, or might have simply contaminated specimens with their intrinsically-high autocrine IFN signatures. We, however, did not observe in any (0/16) of our ccRCC histological samples immune cells in the immediate vicinity of nuclear RelA- or STAT1-positive cells.

The presence of NF-κB and IFN signatures in ccRCC has exciting therapeutic implications, especially as the advanced form of this disease is currently incurable, and as prevalent small-molecule options – centered largely on neutralizing angiogenesis and nutrient-sensing nodes – only delay progression of disease.

First, the prevalence of a constitutive NF-κB signature in ccRCC clinical specimens immediately suggests that NF-κB blockade will have therapeutic benefit in this malignancy. For example, the proteasome inhibitor bortezomib (PS-341, Velcade) mediates its anti-tumor effects partly by preventing NF-κB activation, and pre-clinical studies have shown that bortezomib sensitizes ccRCC cell lines to several anti-neoplastic agents by inhibiting NF-κB [14]. In agreement, we find that expression of *IKBKB* (encoding IKK-β, the key kinase in the canonical NF-κB pathway), is a significant indicator of survival outcome in ccRCC. These data provide rationale for including bortezomib, IKK inhibitors, SMAC mimetics, or other NF-κB pathway blockers as part of combinatorial regimens for advanced ccRCC.

Second, we have identified a subset of NF-κB target effectors whose elevated expression correlates with poor prognosis and reduced overall survival rates: *MMP9, PSMB9*, and *SOD2*. Two of these (*MMP9* and *SOD2*) encode potentially-druggable enzymes (the matrix metalloproteinase MMP9, and the mitochondrial antioxidant MnSOD) that we suggest represent rational targets for second-generation therapeutic strategies in ccRCC.

Third, our discovery of an interferon signature in ccRCC offers opportunities for immunotherapies and IFN-based therapeutics, as the ccRCC interferon signature includes several genes involved in antigen presentation and immune cell recruitment (e.g. *HLA* genes, *B2M*, several chemokine genes, Table S4).

In sum, we report the existence of gene-expression signatures indicative of elevated NF-κB and interferon activity in ccRCC. These signatures correlate well with VHL status, supporting the idea that VHL loss is at least partly responsible for elevated NF-κB/interferon signaling. Both NF-κB and interferon signatures have therapeutic relevance: NF-κB is inhibitable by bortezomib, as well as by other small-molecule agents currently in clinical trials, and constitutive IFN expression may benefit immune-based approaches. Important areas for future research include the development of inhibitors specifically targeting downstream effectors of NF-κB; in particular, enzymes like MMP9 and MnSOD whose expression is significantly correlated with poor prognosis and survival outcome in ccRCC. We expect that such an approach will selectively inhibit the pro-tumorigenic functions of NF-κB, without the widespread side-effects arising from upstream NF-κB blockade.

## Supporting Information

Checklist S1 **Checklist displaying location in the manuscript where individual PRISMA-compliance queries are addressed.**
(DOC)

Figure S1 **Flowchart outlining study methodology.**
(PDF)

Table S1 **Datasets used in this study.**
(XLS)

Table S2 **List of NF-κB target genes used for meta-analysis.**
(XLS)

Table S3 **The ccRCC NF-κB gene signature sorted by category.**
(XLS)

Table S4 **Genes comprising the **ccRCC** IFN signature.**
(XLS)

Table S5 **Summary statistics from Cox PH and AFT methods.**
(DOC)
