# Supplementary material for: Meta-Analysis Identifies NF-κB as a Therapeutic Target in Renal Cancer
Source: PLoS One. 2013 Oct 7;8(10):e76746. doi: 10.1371/journal.pone.0076746 (PMC3792024; doi:10.1371/journal.pone.0076746)
Supplement: Figure S1 — Flowchart outlining study methodology. (PDF) [file pone.0076746.s002.pdf]

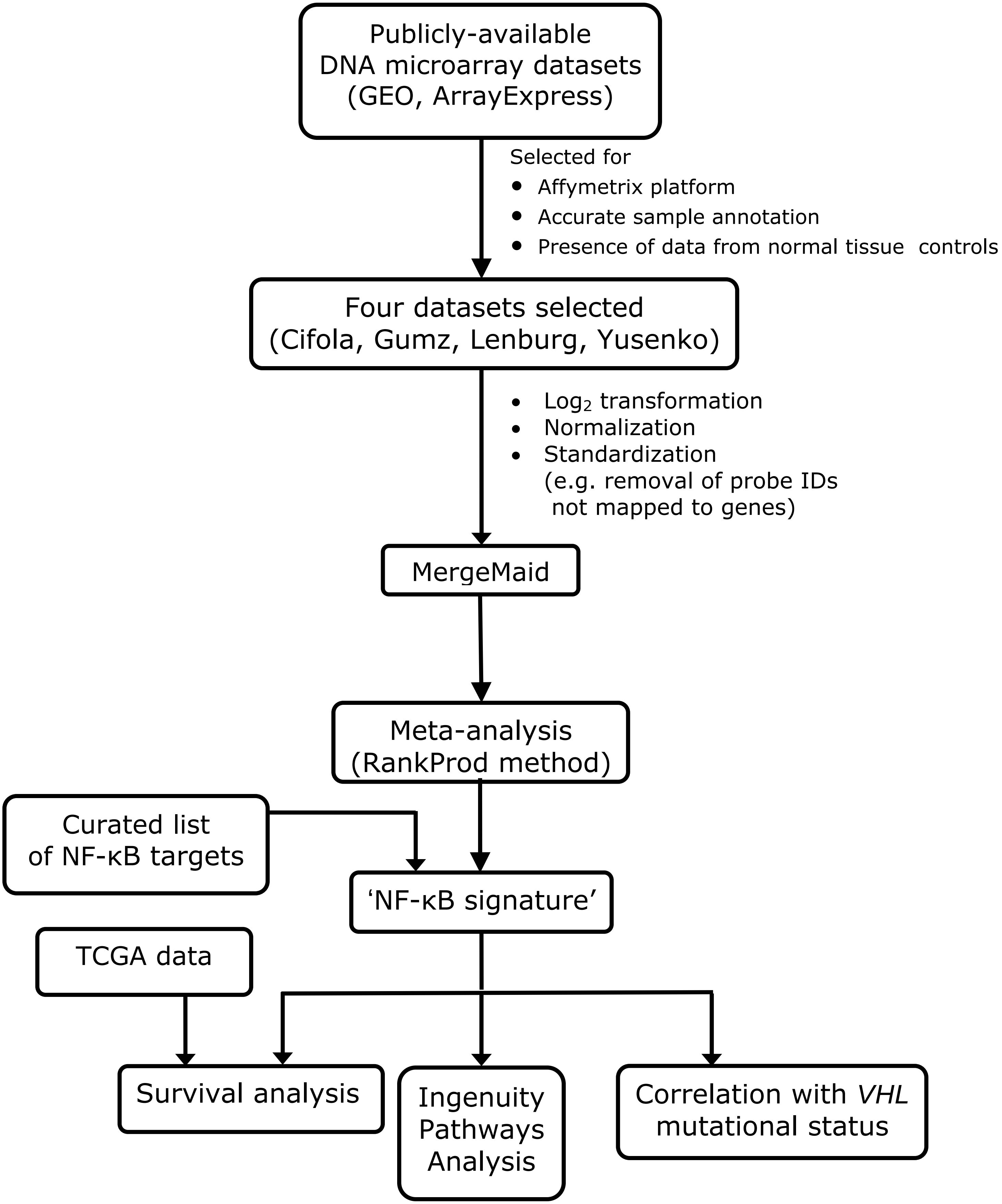
Publicly-available
DNA microarray datasets
(GEO, ArrayExpress)
Selected for
Affymetrix platform
Accurate sample annotation
Presence of data from normal tissue controls
Four datasets selected
(Cifola, Gumz, Lenburg, Yusenko)
Log2 transformation
Normalization
Standardization
(e.g. removal of probe IDs
not mapped to genes)
MergeMaid
Meta-analysis
(RankProd method)
Curated list
of NF-κB targets
'NF-κB signature'
TCGA data
Survival analysis
Ingenuity
Pathways
Analysis
Correlation with VHL
mutational status
