# Supplementary material for: Meta-Analysis Identifies NF-κB as a Therapeutic Target in Renal Cancer
Source: PLoS One. 2013 Oct 7;8(10):e76746. doi: 10.1371/journal.pone.0076746 (PMC3792024; doi:10.1371/journal.pone.0076746)
Supplement: Table S5 — Summary statistics from Cox PH and AFT methods. (DOC) [file pone.0076746.s007.doc]

| **Gene** | **Cox PH *p*-value** | **RR** | **GOF test of Cox PH** | **AFT *p*-value** | **β** |
| --- | --- | --- | --- | --- | --- |
| *MMP9* | 0.030 | 1.50 | 0.35 | 0.150 | -6.88 |
| *PSMB9* | 0.049 | 2.82 | 0.91 | 0.004 | -34.53 |
| *IKBKB* | 0.031 | 7.27 | 0.24 | 0.047 | -46.32 |
| *SOD2* | 0.078 | 1.65 | 0.84 | 0.035 | -15.89 |

A *p*-value threshold of 0.05 by the Cox PH model or by the AFT model was used to determine statistical significance. RR and β quantify the magnitude of the effect of each gene’s expression on overall survival. Please see Methods section for details.
